# Upper extremity open fractures in hospitalized road traffic accident patients: adult versus pediatric cases

**DOI:** 10.1186/s13018-017-0657-1

**Published:** 2017-10-24

**Authors:** Guy Rubin, Kobi Peleg, Adi Givon, Nimrod Rozen

**Affiliations:** 1 Orthopaedic Department, Emek Medical Center, Afula, Israel; 2 Faculty of Medicine, Technion, Haifa, Israel; 3 National Center for Trauma & Emergency Medicine Research, Gertner Institute for Epidemiology and Health Policy Research, Sheba Medical Center, Tel Hashomer, Israel

**Keywords:** Adult, Bicycle, Car, Epidemiology, Open fracture, Motorcycle, Pedestrian, Pediatric, Road traffic accident, Upper extremity

## Abstract

**Background:**

Fractures in pediatrics show epidemiological characteristics which are different from fractures in adults. The objective of this study was to examine the injury profiles of open upper extremity fractures (UEFs) in all modes of injury related to road traffic accidents (RTAs) in adult and pediatric hospitalized patients.

**Methods:**

Data on 103,465 RTA patients between 1997 and 2013 whose records were entered in a centralized country trauma database were reviewed. Data on open UEFs related to mode of injury (car, motorcycle, bicycle, and pedestrian) was compared between adult (18+ years) and pediatric (0–17 years) RTA patients.

**Results:**

Of 103,465 RTA cases, 17,263 (16.7%) had UEFs. Of 73,087 adults, 13,237 (18.1%) included UEFs and of 30,378 pediatric cases, 4026 (13.2%) included UEFs (*p* < 0.0001). Of 17,263 cases with UEFs, we reviewed 22,132 fractures with 2, 743 (12.4%) open fractures. Adults had a greater risk for open fractures (2221, 13%) than the pediatric cases (522, 10.3%) (*p* < 0.0001). Overall, of a total of 22,132 UEFs, most of the fractures were in the radius (22.8%), humerus (20.3%), clavicle (17.5%), and ulna (15.4%). The adult pedestrian group had a significantly higher risk for open UEFs than the pediatric group (11 vs 8%, *p* = 0.0012).

**Conclusions:**

This study demonstrates the difference between adult and pediatric open fractures in hospitalized RTAs. We showed that adults had a greater risk for open UEFs compared to children, and the adult pedestrian group particularly had a significantly higher risk for open UEFs than the pediatric group.

## Background

Fractures in children show epidemiological characteristics which are different from fractures in adults. The epidemiology of adult and pediatric fractures was studied in Edinburgh, Scotland, where it was found that the incidence of fractures in children was almost twice that of fractures in adults. Equal numbers of male and female adults were affected, but there is a strong male predominance in pediatric cases. More adults present with multiple and open fractures. Another difference is that children present mainly with upper limb fractures and have relatively few lower limb fractures [1, 2].

In recent studies on upper extremity fractures (UEFs) in road traffic accident (RTA) hospitalized adult and pediatric patients [3, 4], we found that the fracture rate in children is less than that in adults. Of the total number of pedestrian accidents, about 22% of the children sustained fractures, while 40% of the adults sustained fractures in the same type of accident. The rate of UEFs in the pediatric population was 13.2% compared to 19.9% in the adult group. Multiple UEFs were similar between the groups (23% in the pediatric group and 22% in the adult group). Associated injuries to UEFs were found in about half of our group (56%) but in most of our adult population group (76%). The most frequent UEFs in our pediatric group were similar to our adult group and included the forearm, humerus, and clavicle.

The aim of this study was to identify the epidemiology of RTA open UEFs and to define any commonly occurring patterns. This study focused on all modes of injury related to RTAs (car, motorcycle, bicycle, pedestrian), regarding fracture pattern and age difference.

## Methods

RTA patients (car, motorcycle, bicycle, and pedestrian) in Israel between 1997 and 2013 were reviewed. Data were obtained from the Israel National Trauma Registry (ITR), maintained by Israel’s National Center for Trauma and Emergency Medicine Research at the Gertner Institute for Epidemiology and Health Policy Research. The ITR does not collect data on individuals who were dead at the scene or upon arrival at the hospital, nor does it include patients discharged to home from the Emergency Department. Data are recorded by trained medical registrars at each hospital, and electronic files are transferred to the ITR. During the study period, the ITR included trauma patients admitted to all six level I trauma centers and up to 13 regional trauma centers in Israel.

Medical diagnosis classifications were from the International Classification of Diseases, Ninth Revision, Clinical Modification.

Data included diagnosis of open UEF and type of UEF among different RTA modes. These characteristics were compared between adult (18+ years) and pediatric (0–17 years) RTA patients.

Statistical analysis was performed using SAS statistical software version 9.2 (SAS, Cary, NC). Statistical tests performed included the chi-square test and binomial proportions test. A *p* value less than 0.05 was considered as statistically significant.

Ethical approval was not necessary since the data was collected from a national data registry without patient details.

### Source of funding

There was no external funding source.

## Results

Of 103,465 RTA cases recorded in the ITR in 1997–2013, 17,263 (16.7%) had UEFs. Of 73,087 adults, 13,237 (18.1%) included UEFs and of 30,378 pediatric cases, 4026 (13.2%) included UEFs (*p* < 0.0001) (Table 1).

**Table 1:** Total number of patients and distribution of RTA type among UEF patients

	Car	Motorcycle	Bicycle	Pedestrian	Total
Total patients	47,176	13,087	16,319	26,883	103,465
No. 0–17 patients	6,842	1,192	10,350	11,994	30,378
No. 18+ patients	40,334	11,895	5,969	14,889	73,087
No. (%) UEFs patients	6,459 (37)	3,463 (20)	3,389 (20)	3,952 (23)	17,263
No. (%) UEFs 0–17	803 (20)	217 (6)	1,906 (47)	1,100 (27)	4,026
No. (%) UEFs 18+	5,656 (43)	3,246 (24)	1,483 (11)	2,852 (22)	13,237

Of 17,263 cases with UEFs we reviewed, there were 22,132 fractures with 2, 743 (12.4%) open fractures. Adults had a greater risk for open fractures (2221, 13%) than the children (522, 10.3%) (*p* < 0.0001).

### Fracture type

**Fig. 1:**
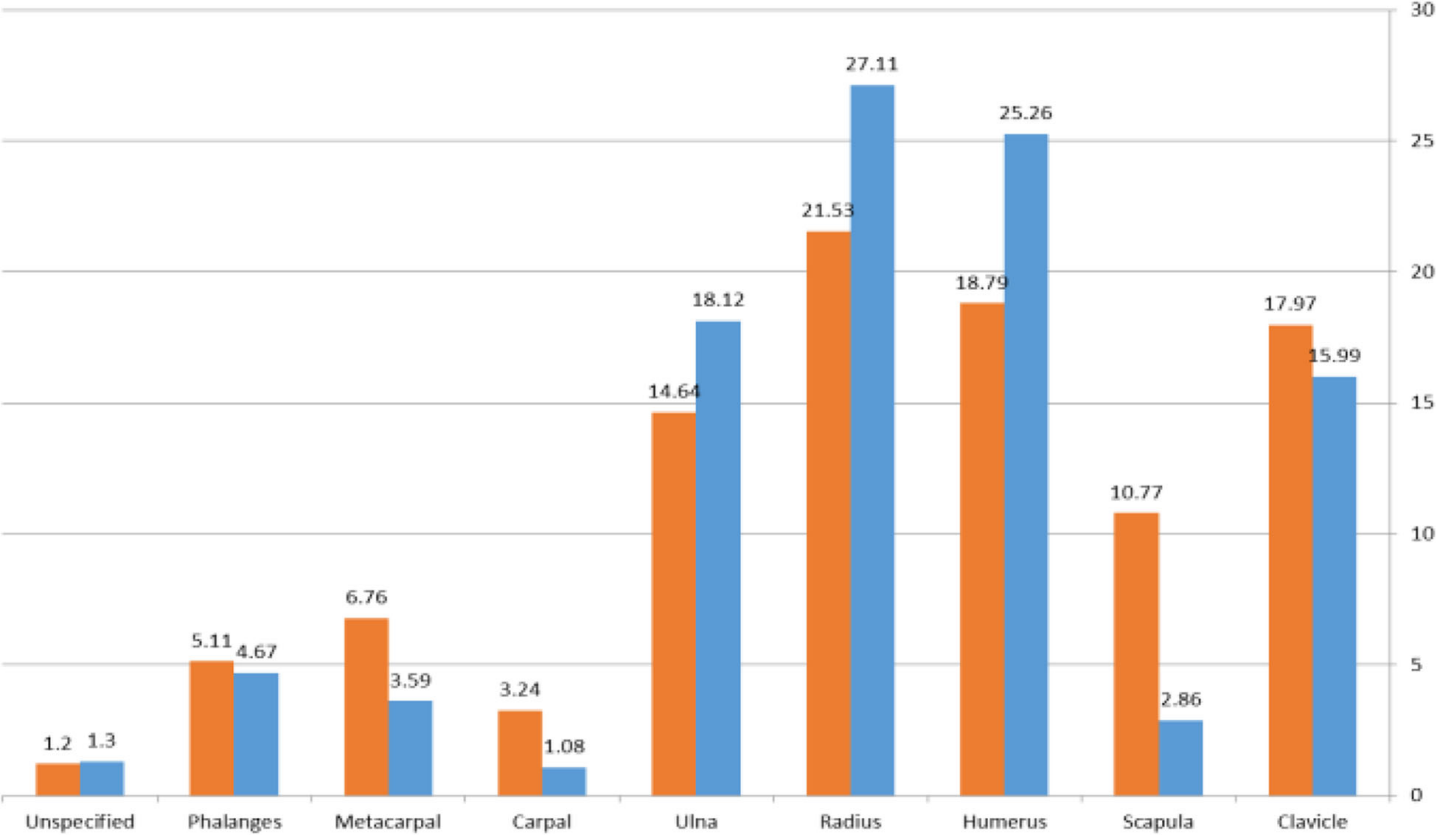
Distribution of fracture type among open UEFs

Overall, of a total of 22,132 UEFs, most of the fractures were in the radius (22.8%), humerus (20.3%), clavicle (17.5%), and ulna (15.4%). The adult group had a similar distribution of fracture type, but the pediatric group had more ulna fractures than clavicle fractures (Fig. 1).

**Fig. 2:**
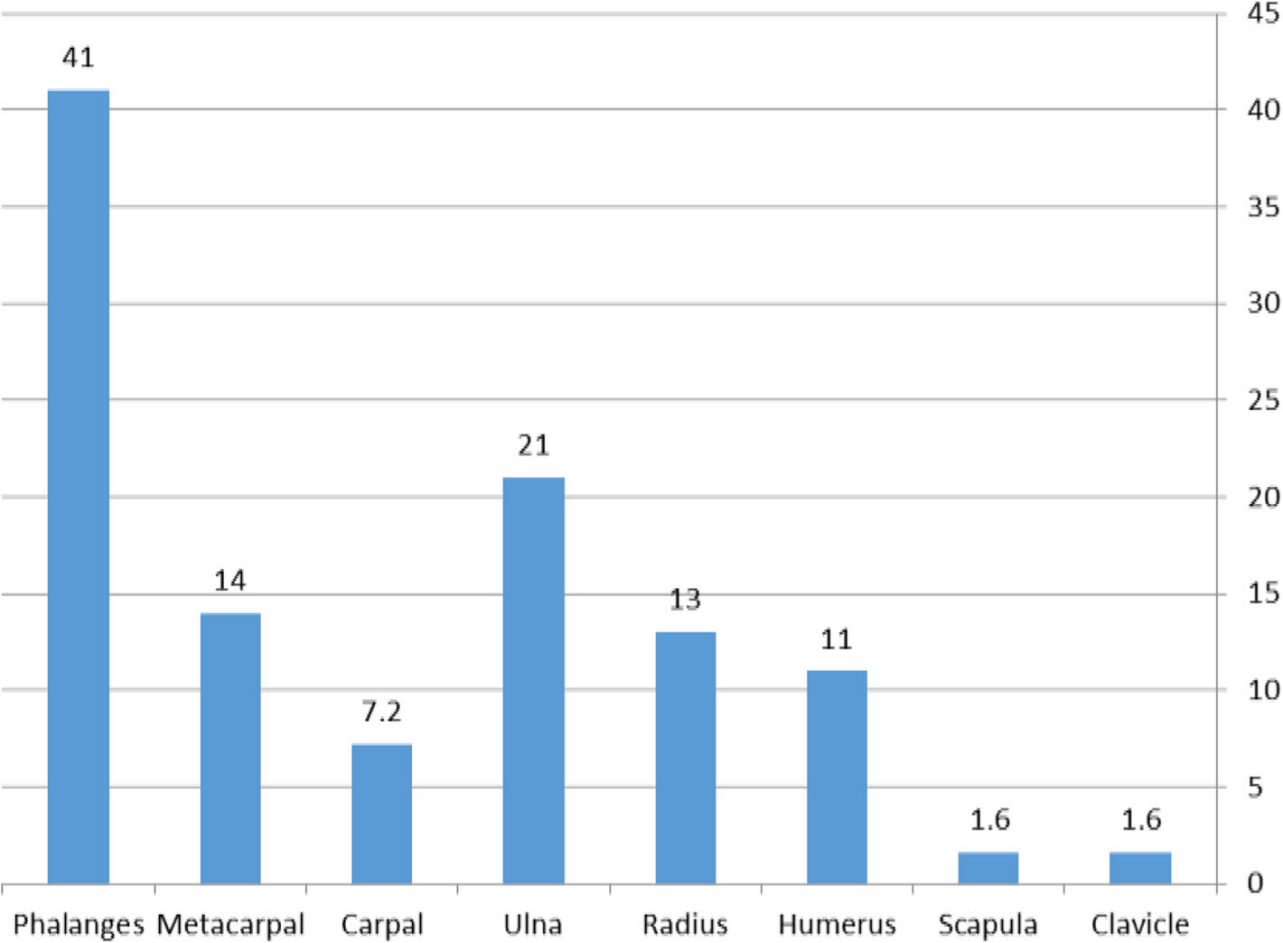
Percent of open fracture among each type of fracture

The percent of open fracture according to fracture type demonstrated that the risk for open fractures in the phalanges was 41%, followed by the ulna (21%), metacarpals (14%), and the radius (13%) (Fig. 2).

### Mode of injury

Of 17,263 cases with UEFs we reviewed, 6459 (37%) were in car occupants, 3463 (20%) in motorcycle riders, 3389 (20%) in bicycle riders, and 3952 (23%) in pedestrians.

Of 22,132 UEFs we reviewed, there were 2743 (12.4%) open fractures with 1198 (14%) in car occupants, 589 (13%) in motorcycle riders, 473 (11%) in bicycle riders, and 483 (10%) in pedestrians. The adult pedestrian group had a significantly higher risk for open UEFs than the pediatric group (11 vs 8%, *p* = 0.0012) (Table 2).

**Table 2:** Percent of patients with UEF from total patients in each type of RTA

	Car	Motorcycle	Bicycle	Pedestrian	Total
Total UEFs	8,339	4,646	4,324	4,823	22,132
No. (%) open UEFs	1,198 (14)	589 (13)	473 (11)	483 (10)	2,743 (12)
No. (%) 0–17 open UEFs	138 (13.5)	26 (9)	260 (10.5)	98 (8)	522 (10)
No. (%) 18+ open UEFs	1,060 (14.5)	563 (13)	213 (12)	385 (11)	2,221 (13)
*p* value 0–17 vs 18+	0.4237	0.0656	0.2182	0.0012	

The risk for each open fracture according to mode of injury and age group is presented in Table 3. In *car occupants*, the most frequent open fractures in adults were ulna (25%), radius (24%), and humerus (21%), compared to pediatrics with radius (26%), phalanges (25%), and ulna (23%). In *motorcycle riders*, the most frequent open fractures in adults were ulna (31%), radius (27%), and phalanges (17%), compared to pediatrics with radius (35%), ulna (31%), and phalanges (23%). In *bicycle riders*, the most open fractures in adults were ulna (32%), radius (25%), and phalanges (15%), compared to pediatrics with radius (30%), ulna (28%), and phalanges (20%). In *pedestrians*, the most frequent open fractures in adults were humerus (28%), ulna (21%), and radius (18%), compared to pediatrics with humerus (33%), radius (19%), phalanges (18%), and ulna (16%).

**Table 3:** Distribution of fracture type among UEFs in each type of RTA

	Total fractures	Total open	Car				Motorcycle				Bicycle				Pedestrian			
			Total fractures	Total open	18+ open	0–17open	Total fractures	Total open	18+ open	0–17open	Total fractures	Total open	18+ open	0–17open	Total fractures	Total open	18+ open	0–17open
Clavicle	3877	63	1515	21	16	5	784	9	9	0	584	16	12	4	994	17	15	2
					2%	3.60%			2%	0			6%	2%			4%	2%
Scapula	1983	32	775	15	13	2	509	2	2	0	184	3	3	0	515	12	9	3
					1%	1.50%			0.40%	0			1%	0			2%	3%
Humerus	4486	508	1607	241	222	19	497	54	53	1	840	70	30	40	1542	143	111	32
					21%	14%			9%	4%			14%	15%			28%	33%
Radius	5049	673	1823	290	254	36	1183	162	153	9	1308	131	53	78	735	90	71	19
					24%	26%			27%	35%			25%	30%			18%	19%
Ulna	3417	723	1309	301	270	31	673	184	176	8	910	140	67	73	525	98	82	16
					25%	23%			31%	31%			32%	28%			21%	16%
Carpal	607	44	199	13	12	1	269	21	21	0	74	3	2	1	65	7	7	0
					1%	0.70%			4%	0			1%	0.40%			2%	0
Metacarpal	1335	185	530	97	89	8	403	39	37	2	185	17	12	5	217	32	26	6
					8%	6%			7%	8%			6%	2%			7%	6%
Phalanges	1108	459	467	199	164	35	270	103	97	6	190	84	31	53	181	73	55	18
					15%	25%			17%	23%			15%	20%			14%	18%
Unspecified	270	56	114				58				49				49			
Total	22,132	2743	8339	1198	1060	138	4646	589	563	26	4324	473	213	260	4823	483	385	98

## Discussion

Open fractures are relatively uncommon and can be complicated with soft tissue damage and compartment syndrome [5]. A study on fracture epidemiology in Edinburgh, Scotland, in 2007–2008 indicated that 59% of the fractures were in the upper limb and only 2.6% of 5271 fractures were open [1]. Court-Brown et al. analyzed all inpatient and outpatient open fractures in a defined adult (> 15 years) population over a 15-year period. They treated 2386 open fractures in 2206 patients, giving an incidence of 30.7/105/year [6]. Rennie et al. studied the epidemiology of fractures in children (< 16) presenting to hospitals in Edinburgh, Scotland, in 2000. They found 2198 fractures in 2168 patients. The overall incidence was 20.2 fractures/1000/year. A review of fracture location showed that 82.2% were in the upper limb, 17.3% were in the lower limb, and 0.5% were in the pelvis or spine. Only 0.7% of fractures were open [2]. Our study showed that open UEFs as a result of RTAs are common in patients admitted to the hospital, with up to 12% of the fractures. We demonstrated that adults had a greater risk for open UEFs as compared to children (13 vs 10%, *p* < 0.0001).

The number of open fractures according to mode of injury demonstrated up to 14% in car occupants. Richter et al. demonstrated that a total of 16% of all cases of front seat car occupants were open injuries [7]. Court-Brown et al. found that only 22.3% of adult open fractures were caused by RTAs or falls from a height [5]. Of the total number of pedestrians involved in vehicle accidents, about 22% of the children sustained fractures, while 40% of the adults sustained fractures in the same type of accident. This has been attributed to the fact that children are more likely to “bounce” when hit [8]. We found that the adult pedestrian group had a significantly higher risk for open UEFs than the pediatric group (11 vs 8%, *p* = 0.0012).

The highest prevalence for an open UEF according to type of bone in the adult general population occurs in the phalanges followed by the forearm [1]. This data has not been studied in the pediatric population. In our study, we demonstrated that the adult group had a similar distribution of fracture type but the pediatric group had more ulna fractures than clavicle fractures.

The limitations of this study are its retrospective nature and the data concerning only hospitalized patients with no data concerning close fractures, other body parts fractures, open fracture classification, or other soft tissue complications. However, the large cohort study and the recommendation for intravenous antibiotic treatment to open fracture inpatients [9] make this study a valuable source of information on the epidemiology of UEFs in adult and pediatric patients.

## Conclusions

This study demonstrates the difference between adult and pediatric open fractures in hospitalized RTAs. We showed that adults had a greater risk for open UEFs compared to children, and the adult pedestrian group particularly had a significantly higher risk for open UEFs than the pediatric group.

## Abbreviations

RTAs: Road traffic accidents
UEFs: Upper extremity fractures
